# Combination Treatment of Icariin and L-DOPA Against 6-OHDA-Lesioned Dopamine Neurotoxicity

**DOI:** 10.3389/fnmol.2018.00155

**Published:** 2018-05-16

**Authors:** Di-Sheng Lu, Ce Chen, Ya-Xin Zheng, Dai-Di Li, Guo-Qing Wang, Jie Liu, Jingshan Shi, Feng Zhang

**Affiliations:** Key Laboratory of Basic Pharmacology of Ministry of Education and Joint International Research Laboratory of Ethnomedicine of Ministry of Education, Zunyi Medical University, Zunyi, China

**Keywords:** Parkinson’s disease, L-DOPA, dyskinesia, Icariin, combination treatment

## Abstract

Until now, the dopamine (DA) precursor, L-3,4-dihydroxyphenylalanine (L-DOPA), remains the gold standard effective drug therapy for Parkinson’s disease (PD) patients. Nevertheless, long-term chronic L-DOPA administration leads to the drug efficacy loss and severe adverse effects, such as L-DOPA-induced dyskinesia (LID). Icariin (ICA), a flavonoid that is extracted from Epimedium, has been proved to evoke neuroprotection against DA neuronal loss in PD animal models. Here, the present study detected the effects of ICA combined with L-DOPA on 6-hydroxydopamine (6-OHDA)-elicited DA neurotoxicity and L-DOPA-induced motor dysfunction as well. PC12 cells were applied to investigate the combination treatment of ICA and L-DOPA against 6-OHDA-lesioned neurotoxicity. In addition, rat substantia nigral stereotaxic injection of 6-OHDA-induced DA neuronal injury was performed to explore the neuroprotective effects mediated by ICA combined with L-DOPA. The pathological movement triggered by L-DOPA was determined by the abnormal involuntary movements (AIM) scores analysis. In PC12 cells, ICA combined with L-DOPA produced better neuroprotection from 6-OHDA-induced neurotoxicity than ICA or L-DOPA alone treatment. In parkinsonian 6-OHDA lesioned rats, ICA conferred DA neuroprotection as monotherapy and an enhancement benefit of L-DOPA treatment after daily administration of L-DOPA and ICA for 21 days. Moreover, ICA ameliorated the development of LID as evidenced by the lowered AIM scores without affecting L-DOPA-mediated efficacy. Furtherly, ICA attenuated neuroinflammation in 6-OHDA-induced DA neuronal loss and the development of LID *in vivo*. In conclusion, these findings suggest ICA might be a potential promising adjuvant to enhance L-DOPA efficacy and attenuate L-DOPA-produced adverse effects in PD.

## Introduction

Parkinson’s disease (PD) is among the most common neurodegenerative movement disorders characterized by the slow and progressive dopamine (DA) neuronal loss in the substantia nigra (SN) and the subsequent depletion of DA content in the striatum. This is accompanied by the behavioral and motor dysfunctions, such as resting tremor, postural instability and muscular rigidity (Fahn, 2003).

Currently, the DA precursor, L-3,4-dihydroxyphenylalanine (L-DOPA) is the most effective drug therapy for PD (Poewe et al., 2010). Nevertheless, long-term L-DOPA treatment leads to a variety of severe adverse effects, and consequently causes the development of motor fluctuation, such as L-DOPA-induced dyskinesia (LID), which limits the treatment efficacy of PD and weakens the life quality of PD patients (Jankovic, 2005). Furthermore, L-DOPA had little effects on several non-motor PD symptoms, including cognitive impairments, sleep disturbances, dysautonomia and apathy (Encarnacion and Hauser, 2008). A battery range of non-dopaminergic therapy has been implied for PD due to its symptoms diversity but has conferred the limited clinical benefits. Thus, strategies of potential synergistic actions in combination with L-DOPA might provide a novel adjuvant therapy for PD.

Icariin (ICA), a flavonoid that is extracted from Epimedium, is well studied to possess a large number of pharmacological properties, including anti-aging, free radical-scavenging and anti-inflammation (Li et al., 2016). Recent studies demonstrate ICA evoked neuroprotection against brain ischemic injury and neurodegenerative diseases (Xiao et al., 2016). In our previous work, ICA attenuated DA neuronal damage and microglia-induced neuroinflammation both *in vivo* and *in vitro* (Wang et al., 2018). Besides, ICA showed synergistic effects combined with methylprednisolone to attenuate experimental autoimmune encephalomyelitis (EAE) through enhancing anti-apoptotic actions (Wei et al., 2015). Therefore, ICA-mediated neuroprotection attracts an increasing attention.

To widen the potential neuroprotective window of ICA, this study detected the effects of ICA combined with L-DOPA on 6-hydroxydopamine (6-OHDA)-elicited DA neurotoxicity and L-DOPA-induced motor dysfunction as well. PC12 cells were applied to investigate the combination treatment of ICA and L-DOPA against 6-OHDA-lesioned neurotoxicity. In addition, rat SN stereotaxic injection of 6-OHDA-induced DA neuronal injury was performed to explore the neuroprotective effects mediated by ICA in combination with L-DOPA. The pathological movements triggered by L-DOPA were determined by the abnormal involuntary movements (AIM) scores detection. Particularly, these findings might provide a more pronounced therapeutic strategy on extrapyramidal disorders compared to monotherapy.

## Materials and Methods

### Reagents

ICA (purity >98%) was obtained from Nanjing Ze Lang Medical Technology Co., Ltd (Nanjing, China). L-DOPA and 6-OHDA were purchased from Sigma Aldrich (St. Lewis, CA, USA). MTT assay kit was from Beijing Solarbio science and Technology Co., Ltd (Beijing, China). Lactate dehydrogenase (LDH) assay Kit was bought from the Beyotime Institute of Biotechnology (Beijing, China). The standard products of DA and its metabolites, 3,4-dihydroxyphenylacetic acid (DOPAC) and homovanilic acid (HVA) were obtained from the National Institute for Food and Drug Control (Beijing, China). The anti-tyrosine hydroxylase (TH, Catalog No. Ab112) antibody was from Abcam (Cambridge, MA, USA). The anti-Bax (Catalog No. 50599-1-AP), Bcl-2 (Catalog No. 12789-1-AP), COX-2 (Catalog No. 12375-1-AP), IL-1β (Catalog No. 16806-1-AP), TNF-α (Catalog No. 17590-1-AP), β-actin (Catalog No. 20536-1-AP), rabbit IgG (Catalog No. SA00001-2), mouse IgG (Catalog No. SA00001-1) and IgG (Catalog No. SA00001-2) antibodies were obtained from Proteintech Group (Chicago, IL, USA).

### Cells Culture and Treatment

PC12 cells were bought from the Cell Culture Center in the Institute of Basic Medical Sciences of Chinese Academy of Medical Sciences (Beijing, China). Cells were cultured in RPMI-1640 medium containing 5% horse serum, 5% fetal bovine serum and streptomycin/penicillin on an atmosphere with 5% CO_2_ at 37°C (Klymov et al., 2015). PC12 cells were treated with different concentrations of ICA and L-DOPA for 24 h before 6-OHDA administration for another 24 h.

### MTT Assay

PC12 cells were cultured in 1 × 10^5^/well in 96-well plates and incubated in an environment with 5% CO_2_ at 37°C for 24 h. After 6-OHDA, ICA and L-DOPA treatment, the culture supernatant was removed and cells were incubated with MTT (5 g/L) for 4 h at 37°C. Formazan crystals in the cells were solubilized using dimethyl sulfoxide (DMSO) and the absorbance was detected by an automatic plate reader (Clinibio 128C, Austria) within a 490-nm wavelength.

### LDH Assay

PC12 cells were added LDH test working fluid mix. LDH activity was measured by detecting the absorbance at 490 nm after incubation for 30 min in the dark at 25°C.

### Flow Cytometric Assessment of Cell Apoptosis

PC12 cells were washed with cold PBS and resuspended in the binding buffer. FITC-labeled Annexin V (5 μl) and PI (5 μl) were added to PC12 cells followed by the incubation at room temperature in the dark for 15 min. Cell apoptosis was assessed by flow cytometry.

### Animals and Treatment

Male Sprague-Dawley rats (200–250 g) were obtained from the Experimental Animal Center of the Third Military Medical University (Chongqing, China; Specific pathogen-free Grade II; Certificate No. SCXK 2012-0005). Rats were housed in the standard conditions of the maintained temperature (25 ± 2°C), humidity (60 ± 4%) and under 12-h light/12-h dark cycle. The experimental uses of animals were performed in accordance with the National Institute of Health Guideline for the Animal Care and Use of Laboratory Animal and approved by the Animal Care and Use Committee of Zunyi Medical University (Zunyi, China). Animals were anesthetized by 7% chloral hydrate (0.5 ml/100 g, v/w) and mounted on a stereotaxic apparatus (Narishige, USA) coupled with a rat adaptor. Rats received a single 6-OHDA (8 μg in 4 μl saline) unilateral injection into the SN pars compacts on the left side of the brain, followed by the coordinates: 5.2 mm posterior to brahma, 2.2 mm lateral to the midline and 8.0 mm ventral to the surface of the skull. Three weeks later, rats were daily treated with L-DOPA (25 mg/kg, i.p.) and ICA (20 mg/kg, p.o.) for 3 weeks (Mo et al., 2010). ICA was daily given 30 min before L-DOPA treatment. Normal control animals received equivolume injections of saline.

### HPLC Coupled With Electrochemical Detection

Animal striatal levels of DA, DOPAC and HVA were measured by HPLC coupled with electrochemical detection. Rat striatal tissue was sonicated in perchloric acid with the internal standards. Then, the homogenate was centrifuged and an aliquot of the supernatant was directly injected onto HPLC equipped with a C_18_ column (Dionex, Germering, Germany). The mobile phase consisted of tetrahydrofuran, monochloroacetic acid and acetonitrile with sodium octyl sulfate and EDTA. The DA, DOPAC and HVA levels were measured by the comparison of peak height ratio of tissue sample with standards and indicated in the quality of wet weight of tissue (Zhang et al., 2006).

### Rotarod Test

Rotarod test is performed to study the muscular coordination. It contained cylindrical arrangement of thin steel rods. The rods were divided into two parts through compartmentalization to detect two rats at the same time. Upon the training session, the speed was at 10 cycles/min and the cut-off time was 3 min during the 5-min test duration. Before the test started, animals were trained on rods till they stayed on rod at least for the cut-off time. Then, rats were permitted to retain stationary for a while at 0 rpm. The rotation speed was steadily increased to 10 rpm in 20-s interval until animals fell off from rungs. Rat behavior changes were determined and the mean duration time stayed on rod was recorded (Khuwaja et al., 2011).

### AIM Scores Assessment

AIM scores were employed to assess the properties of L-DOPA and ICA on rat behavior changes 1, 7, 14 and 21 days after L-DOPA and ICA treatment, respectively. AIM scores are considered to be comparable to LID assessments in patients with PD (Ostock et al., 2015). Rat behavior was detected and scored every 20 min during the 2-h period. Then, the scores of the three AIM subtypes (axial, limb and orolingual) were summed. For each subtype, the dyskinesia severity was scored by a four-point scale (0 = absence, 1 = presence during less than half of the observation time, 2 = presence for more than half of the observation time, 3 = presence all the times but suppressible by external stimulus, 4 = presence all the times and not suppressible by external stimulus). The total AIM scores were calculated by adding each of the axial, limb and orolingual AIM scores (Xie et al., 2014).

### Forepaw Adjusting Steps Test

Rodent akinesia presents as deficient stepping ability in the side of the body contralateral to brain lesion via the forepaw adjusting steps (FAS) test (Lindenbach et al., 2011). Since L-DOPA reversed the stepping deficits, FAS could be applied to investigate whether an adjunctive treatment was associated with L-DOPA-mediated antiparkinsonian properties (Eskow et al., 2007). To perform the test, rat rear torso and one forelimb were held, when the free forelimb was forced to bear the body weight. Rats were then laterally moved through a table in a steady rate of 90 cm/10 s and the number of adjusting steps taken by each forelimb to compensate for lateral movement was quantified. Every session was composed of six trials per forelimb alternating among directions, in which forehand steps were defined as weight-bearing steps taken towards the body and backhand steps were defined as steps moved away from the body. The stepping results were shown as % intact stepping (lesioned steps/ intact steps). The lower % intact stepping score indicated the greater forelimb akinesia.

### Immunohistochemistry Staining and DA Neuronal Counting in SN

Rat brains were cut into 35-μm transverse free-floating sections via a horizontal sliding microtome. A total of 36 consecutive brain slices through the entire SN was collected and every sixth section was performed for the immunocytochemistry staining (Zhang et al., 2010). DA neurons were identified by an anti-TH antibody. Digital images of SN TH-positive neurons were obtained through an Olympus microscope (Olympus^®^, Tokyo, Japan). A region of interest was established by outlining SN via Optimas^®^ version 6.51 (Media Cybernetics Inc., Rockville, MD, USA). Quantification of DA neurons was assessed by blindly visual counting the number of TH-positive neuronal cell bodies by two investigators and the result was analyzed from the average. Then, the mean values of TH-positive neuronal numbers were deduced by averaging the counts of six sections for each brain.

### Western Blotting

Rat brain SN was collected via the Laser capture microdissection method (Greene et al., 2010). Both brain tissues and cell cultures were washed with cold PBS and lysed in a radioimmunoassay (RIPA) lysis buffer. Subsequently, the lysate was incubated in ice for 30 min and centrifuged at 12,000×*g* for 25 min. The protein concentrations were quantified via BCA assay. Finally, membranes were blocked via 5% non-fat Milk and incubated with the following primary antibodies: β-actin (1: 5000), Bax (1: 1000), Bcl-2 (1: 1000), TH (1: 2000), COX-2 (1: 2000), IL-1β (1: 2000), TNF-α (1: 1000) and horseradish peroxidase-conjugated secondary antibodies (1: 5000). The blot was visualized by the enhanced ECL reagent.

### Statistical Analysis

Results were indicated as mean ± standard error of the mean (SEM). Statistical significance was analyzed by one- or two-way ANOVA through the GraphPad Prism software (GraphPad Software Inc., San Diego, CA, USA). Upon ANOVA demonstrating the significant differences, pairwise comparison between means was evaluated by Bonferroni’s *post hoc* test with correction. A value of *p* < 0.05 was considered statistically significant.

## Results

### ICA Combined With L-DOPA Attenuated 6-OHDA-Induced Cytotoxicity in PC12 Cells

**Figure 1:**
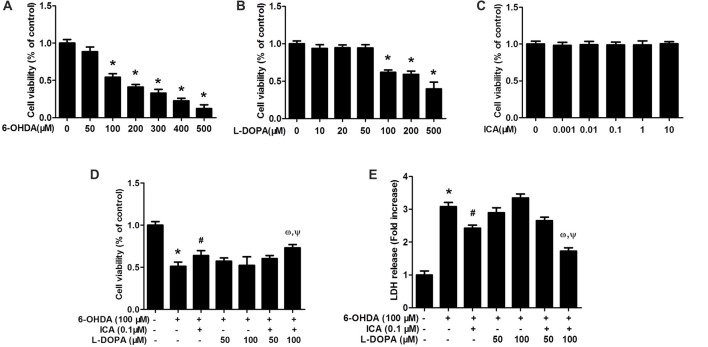
Icariin (ICA) combined with L-3,4-dihydroxyphenylalanine (L-DOPA) protected against 6-hydroxydopamine (6-OHDA)-induced cytotoxicity in PC12 cells. PC12 cells were treated with different concentrations of 6-OHDA **(A)**, L-DOPA **(B)** and ICA **(C)** for 24 h and cell viability was detected by MTT assay. In addition, PC12 cells were pretreated with ICA (0.1 μM) and L-DOPA (50 and 100 μM) for 24 h and then incubated with 6-OHDA (100 μM) for 24 h. Cell viability **(D)** and the intracellular lactate dehydrogenase (LDH) levels **(E)** were determined. Data were shown as mean ± standard error of the mean (SEM) from three independent experiments performed in triplicate. *p < 0.05 compared with the control cultures; ^#^*p* < 0.05 compared with 6-OHDA-treated cultures; ^ω^*p* < 0.05 compared with 6-OHDA+ICA (0.1 μM)-treated cultures; ^Ψ^*p* < 0.05 compared with 6-OHDA+L-DOPA (100 μM)-treated cultures.

PC12 cells were treated with different concentrations of 6-OHDA, L-DOPA and ICA to evaluate the cytotoxicity of 6-OHDA, L-DOPA and ICA. Cell viability assay indicated that both of 6-OHDA and L-DOPA from the concentration of 100 μM significantly reduced PC12 cell viability shown in Figure 1A (*F*_(6,14)_ = 145.2, *p* < 0.0001) and Figure 1B (*F*_(6,14)_ = 59.86, *p* < 0.001), while ICA (0.001–10 μM) did not show any cytotoxicity shown in Figure 1C (*F*_(5,12)_ = 0.1267, *p* > 0.05). In addition, as indicated in Figure 1D (*F*_(6,14)_ = 25.66, *p* < 0.001), ICA (0.1 μM) attenuated 6-OHDA-induced PC12 cell damage and L-DOPA (50–100 μM) had no significant protection against 6-OHDA-lesioned PC cells. However, ICA (0.1 μM) in combination with L-DOPA (100 μM) significantly produced better neuroprotection from 6-OHDA-induced cell injury compared with 6-OHDA+ICA (0.1 μM; *t* = 4.463, *p* < 0.05) and 6-OHDA+L-DOPA (100 μM; *t* = 4.204, *p* < 0.05) treatment. Similarly, the LDH release assay exhibited that 6-OHDA increased LDH release and ICA reduced 6-OHDA-induced increase of LDH release, whereas L-DOPA (50 and 100 μM) had no significant actions on 6-OHDA-increased LDH release shown in Figure 1E (*F*_(6,14)_ = 143.2, *p* < 0.001). Furthermore, the LDH release was lower in ICA combined with L-DOPA (100 μM) group than that in ICA (*t* = 7.174, *p* < 0.01) or L-DOPA (*t* = 16.61, *p* < 0.01) alone treatment after 6-OHDA administration. These results suggested that ICA combined with L-DOPA had more protective effects on 6-OHDA-induced PC12 cell damage than ICA or L-DOPA alone treatment.

### ICA in Combination With L-DOPA Decreased 6-OHDA-Induced PC12 Cell Apoptosis

**Figure 2:**
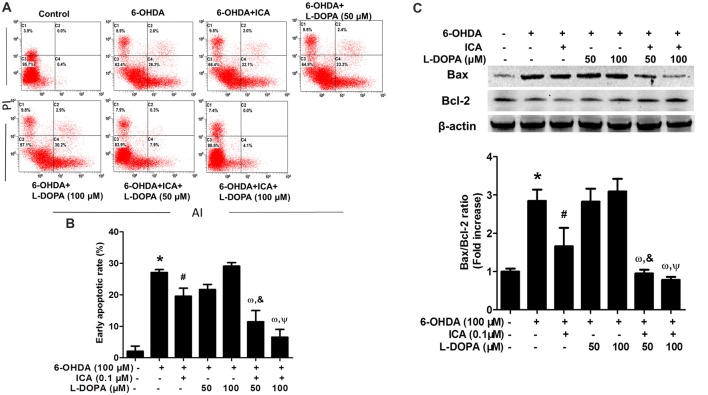
ICA combined with L-DOPA ameliorated 6-OHDA-induced PC12 cell apoptosis. PC12 cells were pretreated with ICA (0.1 μM) and L-DOPA (50 and 100 μM) for 24 h followed by 6-OHDA (100 μM) treatment for 24 h. Flow cytometry analysis **(A)** of 6-OHDA-induced apoptosis in PC12 cells and the quantitative analysis **(B)** of the rate of early apoptotic cells (this population corresponded to quadrant C2 in **(A)** were performed. The protein levels of Bcl-2 and Bax were detected by western blotting **(C)**. Data were shown as mean ± SEM from three independent experiments performed in triplicate. *p < 0.05 compared with the control cultures; ^#^*p* < 0.05 compared with 6-OHDA-treated cultures; ^&^*p* < 0.05 compared with 6-OHDA+L-DOPA (50 μM)-treated cultures; ^ω^*p* < 0.05 compared with 6-OHDA+ ICA (0.1 μM)-treated cultures; ^Ψ^*p* < 0.05 compared with 6-OHDA+L-DOPA (100 μM)-treated cultures.

The properties of ICA and L-DOPA on 6-OHDA-elicited PC12 cell apoptosis were determined by flow cytometry. As shown in Figures 2A,B (*F*_(6,14)_ = 45.78, *p* < 0.001), compared with the control cultures with the early apoptotic rate of 1.5%, the percentage of early apoptosis cells in 6-OHDA group was increased to 26.2% (*t* = 11.62, *p* < 0.01). Compared with 6-OHDA-treated cultures, ICA (0.1 μM) significantly inhibited cell apoptosis (*t* = 4.353, *p* < 0.05) and L-DOPA (50 and 100 μM) had no significantly inhibitory effects on 6-OHDA-induced PC12 cell apoptosis. An interesting evidence indicated that L-DOPA alone (25–100 μM) induced PC12 cell apoptosis (Walkinshaw and Waters, 1995). However, ICA combined with L-DOPA (50 and 100 μM) exhibited more anti-apoptotic properties against 6-OHDA-induced cell apoptosis than ICA or L-DOPA (50 and 100 μM) treatment. Moreover, ICA (0.1 μM) attenuated 6-OHDA-elicited up-regulation of Bax and down-regulation of Bcl-2, thereby decreasing the Bax/Bcl-2 ratio. Furtherly, ICA together with L-DOPA (50 and 100 μM) led to more decrease of Bax/Bcl-2 ratio than ICA or L-DOPA (50 and 100 μM) treatment after 6-OHDA application shown in Figure 2C (*F*_(6,14)_ = 38.67, *p* < 0.001). These results demonstrated that ICA in combination with L-DOPA significantly ameliorated 6-OHDA-induced neurotoxicity.

### ICA Combined With L-DOPA Ameliorated 6-OHDA-Induced DA Neuronal Loss

**Figure 3:**
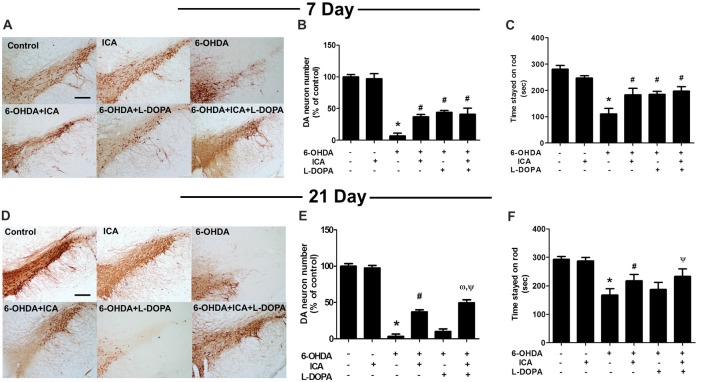
ICA combined with L-DOPA protected dopamine (DA) neurons from 6-OHDA-induced neurotoxicity *in vivo*. Rats received unilateral injection of 6-OHDA (8 μg) in the substantia nigra (SN) pars compacts on the left side of the brain. Three weeks later, rats were administrated L-DOPA (25 mg/kg, i.p.) along with ICA (20 mg/kg, p.o.) daily for 3 weeks. After L-DOPA and ICA treatment for 7 and 21 days, rats were sacrificed and SN DA neurons in the brain sections were recognized with an anti-TH antibody **(A,D)**. Scale bar = 200 μm. DA neuronal lesion in the SN was analyzed via the quantification of TH-positive neurons by immunostaining, respectively **(B,E)**. Rat behavior changes were assessed by rotarod test. The time stayed on the rod was recorded **(C,F)**. Data were shown as mean ± SEM from six rats (*n* = 6). *p < 0.05 compared with the control group; ^#^*p* < 0.05 compared with 6-OHDA group; ^ω^*p* < 0.05 compared with 6-OHDA+ICA group; ^Ψ^*p* < 0.05 compared with 6-OHDA+L-DOPA group.

Rat brain DA neurons were quantified 7 and 21 days after ICA and L-DOPA first treatment, respectively. As shown in Figures 3A,B (*F*_(5,30)_ = 111.8, *p* < 0.001), Figures 3D,E (*F*_(5,30)_ = 446.7, *p* < 0.001), 6-OHDA induced DA neuronal loss and ICA reduced 6-OHDA-lesioned DA neurons from ICA treatment for 7 days, while L-DOPA-mediated DA neuroprotection was shown in L-DOPA treatment for 7 days and disappeared 21 days later. Moreover, ICA+L-DOPA-mediated better DA neuroprotection against 6-OHDA-induced DA neuronal damage than ICA (*t* = 4.557, *p* < 0.01) and L-DOPA (*t* = 14.22, *p* < 0.01) alone treatment was further demonstrated till ICA and L-DOPA treatment for 21 days. Besides DA neuronal quantification, rat behavior changes were also detected via rotarod test. As shown in Figure 3C (*F*_(5,30)_ = 83.40, *p* < 0.001) and Figure 3F (*F*_(5,30)_ = 48.35, *p* < 0.001), ICA (*t* = 7.999, *p* < 0.01) and ICA+L-DOPA (*t* = 9.844, *p* < 0.01) attenuated 6-OHDA-induced decrease of the time rat stayed on rod from ICA and L-DOPA treatment for 7 days but there was no significant difference between ICA and ICA+L-DOPA treatment groups. However, consistent with the effects of L-DOPA on DA neuronal number, L-DOPA-improved 6-OHDA-induced rat behavior changes was only discerned after L-DOPA treatment for 7 days (*t* = 8.240, *p* < 0.01) not for 21 days.

**Figure 4:**
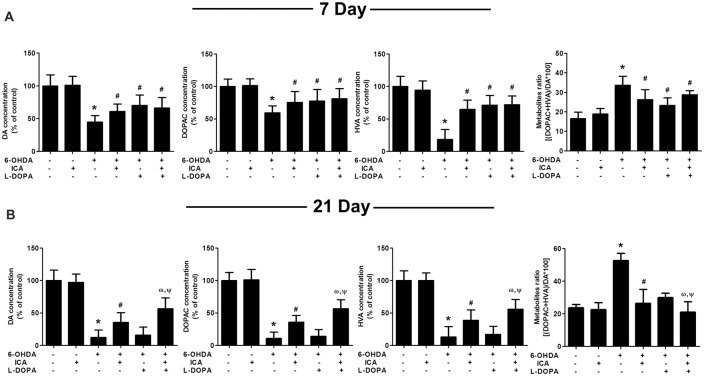
ICA combined with L-DOPA attenuated the decrease of DA and its metabolites in rat striatum treated with 6-OHDA. The striatal tissue levels of DA, 3,4-dihydroxyphenylacetic acid (DOPAC) and homovanilic acid (HVA) were measured by HPLC coupled with electrochemical detection at 7 **(A)** and 21 **(B)** days after the first treatment of L-DOPA and ICA, respectively. Also, the DA metabolite ratio [(HVA + DOPAC)/DA *100] was calculated. Data were shown as mean ± SEM from six rats (*n* = 6). *p < 0.05 compared with the control group; ^#^*p* < 0.05 compared with 6-OHDA group; ^ω^*p* < 0.05 compared with 6-OHDA+ICA group; ^Ψ^*p* < 0.05 compared with 6-OHDA+L-DOPA group.

In addition to DA neuronal number and rat behavior detection, the neuroprotective effects of ICA combined with L-DOPA against 6-OHDA-induced depletion of striatal DA, DOPAC and HVA levels were investigated by HPLC coupled with electrochemical detection. As shown in Figure 4A, ICA, L-DOPA and ICA+L-DOPA attenuated 6-OHDA-decreased DA (*F*_(5,30)_ = 7.448, *p* < 0.01), DOPAC (*F*_(5,30)_ = 3.822, *p* < 0.01) and HVA (*F*_(5,30)_ = 11.63, *p* < 0.01) levels 7 days after ICA and L-DOPA treatment and no significant difference among ICA, L-DOPA and ICA+L-DOPA these three groups was indicated. Moreover, compared with 6-OHDA group, the metabolite ratio [(HVA+DOPAC) *100/DA] in these three groups showed a decreased DA turnover (*F*_(5,30)_ = 5.607, *p* < 0.01). Twenty-one days after ICA and L-DOPA treatment, ICA combined with L-DOPA had better effects on the attenuation of 6-OHDA-elicited decrease of DA (*F*_(5,30)_ = 14.74, *p* < 0.001), DOPAC (*F*_(5,30)_ = 21.34, *p* < 0.001)and HVA (*F*_(5,30)_ = 16.36, *p* < 0.0001) levels and also increase of the metabolite ratio than ICA treatment (*F*_(5,30)_ = 10.25, *p* < 0.001), whereas L-DOPA showed no significant protective effects against 6-OHDA-induced DA and its metabolites changes (Figure 4B).

### ICA Alleviated L-DOPA-Produced Motor Dysfunctions Without Affecting L-DOPA-Mediated Efficacy

**Figure 5:**
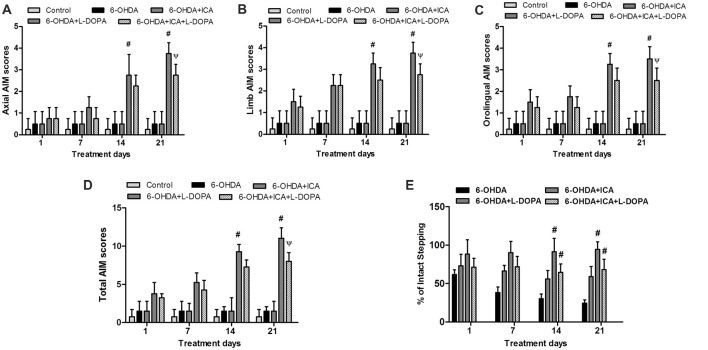
ICA alleviated LID in 6-OHDA-lesioned rats. One, 7, 14 and 21 days after daily L-DOPA (25 mg/kg, i.p.) combined with ICA (20 mg/kg, p.o.) treatment, the effects of ICA on L-OHDA-induced changes of axial **(A)**, limb **(B)** and orolingual **(C)** abnormal involuntary movements (AIM) scores were evaluated. The total AIM scores were also detected **(D)**. The forepaw adjusting steps (FAS) test was applied to determine the effects of L-DOPA together with ICA on forelimb stepping changes **(E)**. Data were shown as mean ± SEM from six rats (*n* = 6). ^#^*p* < 0.05 compared with 6-OHDA-treated group;^Ψ^*p* < 0.05 compared with 6-OHDA+L-DOPA group.

The body AIM scores using the different dyskinesia subtypes (axial, limb and orolingual AIM scores) was analyzed, respectively. In 6-OHDA-lesioned rats, the AIM was gradually aggravated after continuous administration of L-DOPA. In contrast, daily treatment ICA before L-DOPA administration in 6-OHDA-lesioned rats reduced L-DOPA-induced increase of all three subtypes scores 21 days after ICA and L-DOPA treatment shown in Figure 5A (*F*_(4,25)_ = 35.91, *p* < 0.0001), Figure 5B (*F*_(4,25)_ = 73.21, *p* < 0.001) and Figure 5C (*F*_(4,25)_ = 27.11, *p* < 0.0001). Taken together, ICA significantly attenuated L-DOPA-induced development of total AIM scores at ICA and L-DOPA treatment for 21 days shown in Figure 5D (*F*_(4,25)_ = 69.24, *p* < 0.001). These results implied that ICA could alleviate LID. In addition, to determine whether ICA treatment altered L-DOPA-mediated anti-parkinsonian efficacy, rats were treated with the anti-dyskinetic dose of ICA followed by L-DOPA and the motor performance was detected by the FAS test. As shown in Figure 5E, L-DOPA significantly reversed 6-OHDA-induced stepping deficits and ICA combined with L-DOPA maintained this anti-parkinsonian effect compared with 6-OHDA group 21 days after ICA and L-DOPA treatment (*F*_(3,20)_ = 62.37, *p* < 0.01).

### ICA Combined With L-DOPA Inhibited Inflammatory Factors Expressions in 6-OHDA-Treated SN

**Figure 6:**
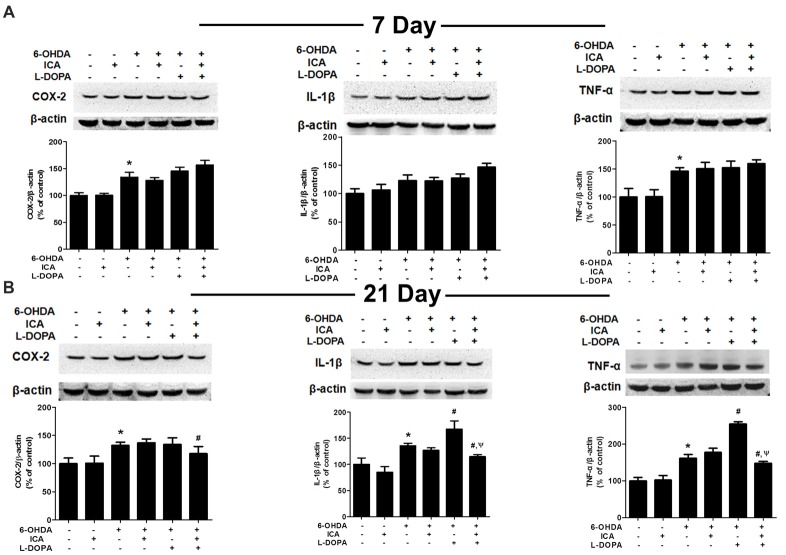
ICA inhibited the neuroinflammation in LID after 6-OHDA-induced DA neuronal damage. The protein levels of neuroinflammatory factors, such as COX-2, IL-1β and TNF-α, in the SN were detected 7 **(A)** and 21 **(B)** days after daily L-DOPA (25 mg/kg, i.p.) combined with ICA (20 mg/kg, p.o.) treatment by western blotting. Data were shown as mean ± SEM from six rats (*n* = 6). *p < 0.05 compared with the control group; ^#^*p* < 0.05 compared with 6-OHDA-treated group; ^ψ^*p* < 0.05 compared with 6-OHDA+L-DOPA group.

Inflammatory factors expressions in the SN were measured via western blotting. Compared with the control group, the COX-2 (*F*_(5,30)_ = 35.58, *p* < 0.001) and TNF-α (*F*_(5,30)_ = 18.27, *p* < 0.0001) protein expressions in 6-OHDA group were significantly increased 7 days after ICA and L-DOPA treatment, while no higher IL-1β (*F*_(5,30)_ = 12.25, *p* < 0.01) protein expression was shown. However, ICA, L-DOPA and ICA+L-DOPA had no significant effects on 6-OHDA-increased inflammatory factors expressions (Figure 6A). On contrary, ICA combined with L-DOPA reduced 6-OHDA-induced increase of COX-2 (*F*_(5,30)_ = 7.968, *p* < 0.01), IL-1β (*F*_(5,30)_ = 26.15, *p* < 0.001) and TNF-α (*F*_(5,30)_ = 76.78, *p* < 0.0001) protein expressions 21 days after ICA and L-DOPA treatment, whereas L-DOPA still increased IL-1β (*t* = 3.993, *p* < 0.05) and TNF-α (*t* = 10.12, *p* < 0.01) protein expressions after 6-OHDA treatment (Figure 6B).

## Discussion

This present study indicated that ICA conferred DA neuroprotection as monotherapy and an enhancement benefit of L-DOPA treatment after daily administration of L-DOPA and ICA for 21 days in parkinsonian 6-OHDA lesioned rats. Additionally, ICA ameliorated the development of LID as evidenced by the lowered AIM scores without affecting L-DOPA-mediated efficacy via FAS test. Furthermore, ICA attenuated neuroinflammation in the development of LID *in vivo*. Collectively, these results suggested ICA might possess as an adjunct treatment to enhance the efficacy of L-DOPA and ameliorate LID in PD (Supplementary Figure S1).

L-DOPA holds the gold standard therapy for PD patients. However, its long-term administration resulted in severe motor complications known as LID, which severely limits the efficacy of current treatments of PD (Bordet et al., 2000). It is reported that LID influences nearly 80% of PD patients after L-DOPA treatment for 5–10 years and some of these PD patients have to stop this treatment because of serious LID (Shin et al., 2015). Although the lowered dose of L-DOPA is one strategy to attenuate the adverse effects induced by L-DOPA (Ahn et al., 2017), the efficacy could be highly decreased (Kurlan, 2005). Thus, to explore the combination treatment to compensate for this limitation might provide a new scenario for PD. ICA is a natural component confirmed to have anti-aging, anti-oxidant and anti-inflammatory activities (Liu et al., 2015). It has been evaluated that ICA protected against 1-methyl-4-phenyl-1,2,3,6-tetrahydropyridine (MPTP)-induced DA neuronal damage through the regulation of phosphatidylinositol 3-kinase (PI3K) and mitogen-activated protein kinase (MEK) signaling pathway (Chen et al., 2017). Our previous study found that ICA attenuated 6-OHDA and lipopolysaccharide (LPS)-induced DA neuronal death and thereby improved motor dysfunction (Wang et al., 2018). In this study, ICA enhanced L-DOPA-mediated DA neuroprotection and ameliorated L-DOPA-induced motor dysfunction without interfering with the therapeutic efficacy of L-DOPA, suggesting that the use of the potential neuroprotective agent in combination with available drugs might serve as promising therapeutic potential for PD treatment.

In addition, it is known that dyskinesia occurs only upon dopaminergic treatment and the presence of DA neuronal loss in the SN (Rascol and Fabre, 2001). Nevertheless, the pathogenesis of LID remains poorly understood at present. Over the last decade, concerns regarding L-DOPA-induced neurotoxicity and its potential role on PD progression and the development of L-DOPA-induced motor dysfunctions have been raised (Lipski et al., 2011). In PD pathogenesis, neuroinflammation is recognized to be one of the main contributors and might be amplified by the increased DA metabolism and L-DOPA during the continuous L-DOPA treatment. However, the contribution of such event to the development of motor side complications is incompletely elucidated. Recently, multiple lines of evidence have implied a possible contribution of L-DOPA-elicited neuroinflammatory responses to LID (Carta et al., 2017). Since preclinical studies support the role of neuroinflammation on LID, whether an exacerbated neuroinflammation might be involved in LID development of PD patients could be unilluminated until now. However, during PD patients with L-DOPA treatment in late disease stage, it is not excluded that the neuroinflammatory reactions participate in this treatment. The mechanisms how L-DOPA would promote the neuroinflammatory reactions, and how that would in turn influence the dyskinetic outcomes, warrants further investigation. In this *in vivo* study, ICA combined with L-DOPA decreased 6-OHDA-induced protein expressions of inflammatory factors 21 days not 7 days after ICA and L-DOPA treatment, which implied that the inhibition of neuroinflammation might be one of the contributors to ICA-attenuated LID. In parallel with our previous work, we found that ICA inhibited microglia-mediated inflammation in *in vivo* and *in vitro* studies (Wang et al., 2018). However, we couldn’t rule out other factors, such as the attenuation of mitochondrial oxidative stress, participated in the mechanisms underlying ICA-mediated DA neuroprotection and attenuation of LID. Since the pathogenesis of PD and LID was still unelucidated, neuroinflammation might be involved in the process of PD and LID and the corresponding mechanisms were still not clear. This present study was the pilot investigation to indicate that ICA enhanced L-DOPA-mediated DA neuroprotection and attenuated L-DOPA-induced dyskinetic actions. Thus, the mechanisms underlying ICA-mediated these beneficial effects require deep rigorous exploration.

At present, no effective treatment is available to halt PD progression. Although L-DOPA is the gold standard treatment for PD, it is frequently related to a series of side complications and unsatisfactory outcomes. Therefore, to develop the effective treatment avenues to stop the progression of PD is of paramount importance (AlDakheel et al., 2014). So far, critics have argued that neuroprotective agents couldn’t fulfill the therapeutic window for PD, since patients diagnosed as PD already present more than 50–60% dopaminergic deficits in the SN (Lang et al., 2013). This study indicated that ICA conferred synergistic effects with L-DOPA against DA neuronal loss and alleviated LID. Despite this optimistic perspective for the future use of ICA potential treatment for PD, most of the findings are determined in one type of PD animal model. Therefore, to verify the translational value of ICA, this combination treatment need be rigorously corroborated in other PD animal models.

## Conclusion

This study demonstrates that ICA has synergistic effects against DA neuronal loss combined with L-DOPA and anti-dyskinetic actions induced by L-DOPA. These findings suggest ICA might be a potential promising adjuvant to enhance L-DOPA efficacy and attenuate L-DOPA-produced adverse effects in PD.

## Author Contributions

FZ conceived and designed the experiments. D-SL, CC and Y-XZ participated in the experiments performance and G-QW, D-DL, JL, JS and FZ finished data analysis. FZ wrote and revised the manuscript. All the authors checked the contents of the manuscript, validated the accuracy of the data and approved the submitted manuscript.

## Conflict of Interest Statement

The authors declare that the research was conducted in the absence of any commercial or financial relationships that could be construed as a potential conflict of interest.

## Supplementary Material

FIGURE S1 Icariin enhances L-DOPA efficacy and attenuates L-DOPA-produced adverse effects in Parkinson’s disease.
