# Corrigendum: Combination Treatment of Icariin and L-DOPA Against 6-OHDA-Lesioned Dopamine Neurotoxicity

**DOI:** 10.3389/fnmol.2020.605722

**Published:** 2020-10-29

**Authors:** Di-Sheng Lu, Ce Chen, Ya-Xin Zheng, Dai-Di Li, Guo-Qing Wang, Jie Liu, Jingshan Shi, Feng Zhang

**Affiliations:** Key Laboratory of Basic Pharmacology of Ministry of Education and Joint International Research Laboratory of Ethnomedicine of Ministry of Education, Zunyi Medical University, Zunyi, China

**Keywords:** Parkinson's disease, L-DOPA, dyskinesia, Icariin, combination treatment

In the original article, there was a mistake in Figure 6 as published. One western blot band of β-actin corresponding to TNF-α protein level 21 days after daily L-DOPA combined with ICA treatment in Figure 6B was inserted incorrectly. The corrected Figure 6 appears below.

**Figure 6:**
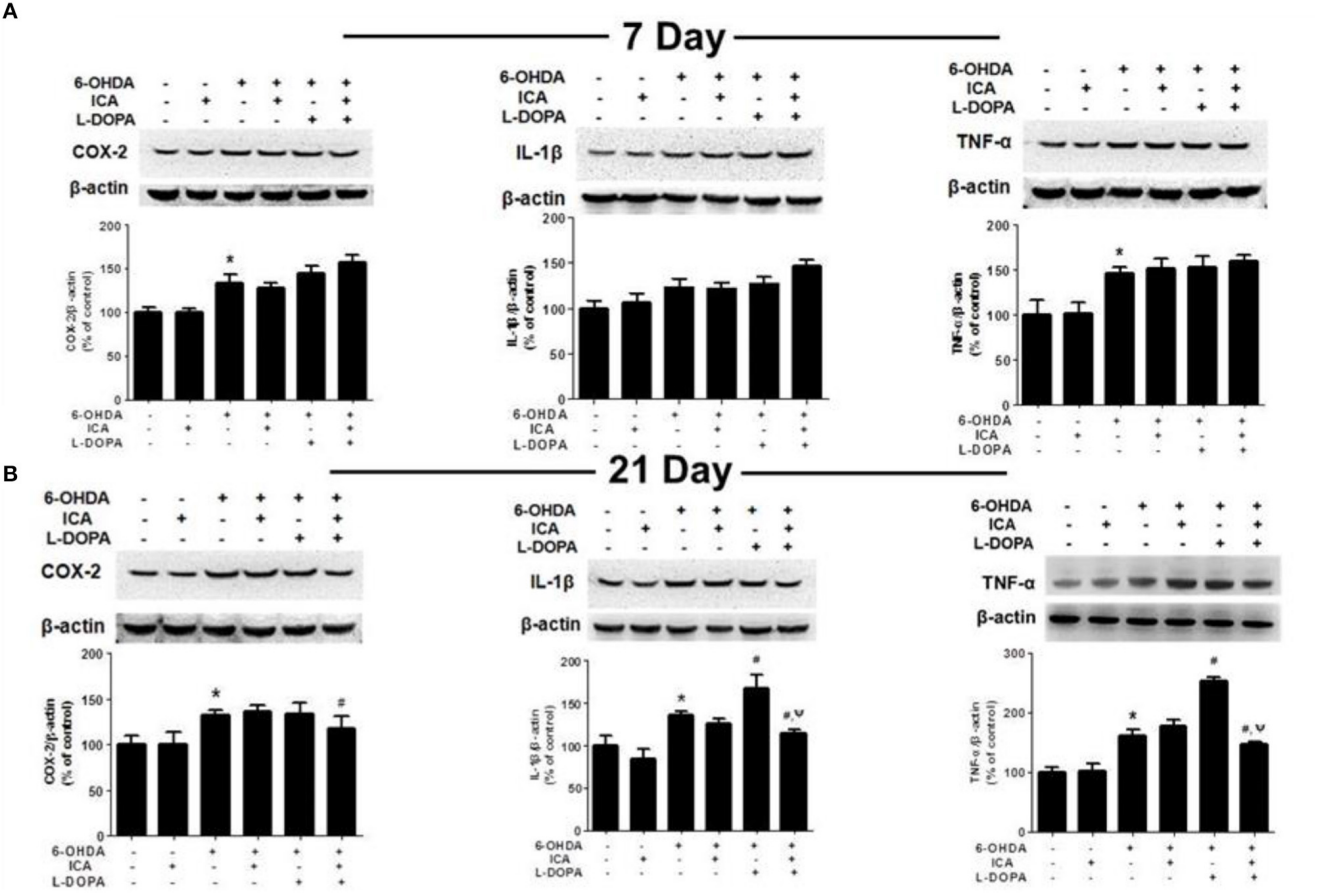
ICA inhibited the neuroinflammation in LID after 6-OHDA-induced DA neuronal damage. The protein levels of neuroinflammatory factors, such as COX-2, IL-1β and TNF-α, in the SN were detected 7 **(A)** and 21 **(B)** days after daily L-DOPA (25 mg/kg, i.p.) combined with ICA (20 mg/kg, p.o.) treatment by western blotting. Data were shown as mean ± SEM from six rats (*n* = 6). *p <0.05 compared with the control group; ^#^*p* <0.05 compared with 6-OHDA-treated group; ^Ψ^*p* <0.05 compared with 6-OHDA+L-DOPA group.

The authors apologize for this error and state that this does not change the scientific conclusions of the article in any way. The original article has been updated.

