# Supplementary figures and images for: Combination Treatment of Icariin and L-DOPA Against 6-OHDA-Lesioned Dopamine Neurotoxicity
Source: Front Mol Neurosci. 2018 May 16;11:155. doi: 10.3389/fnmol.2018.00155 (PMC5964195; doi:10.3389/fnmol.2018.00155)

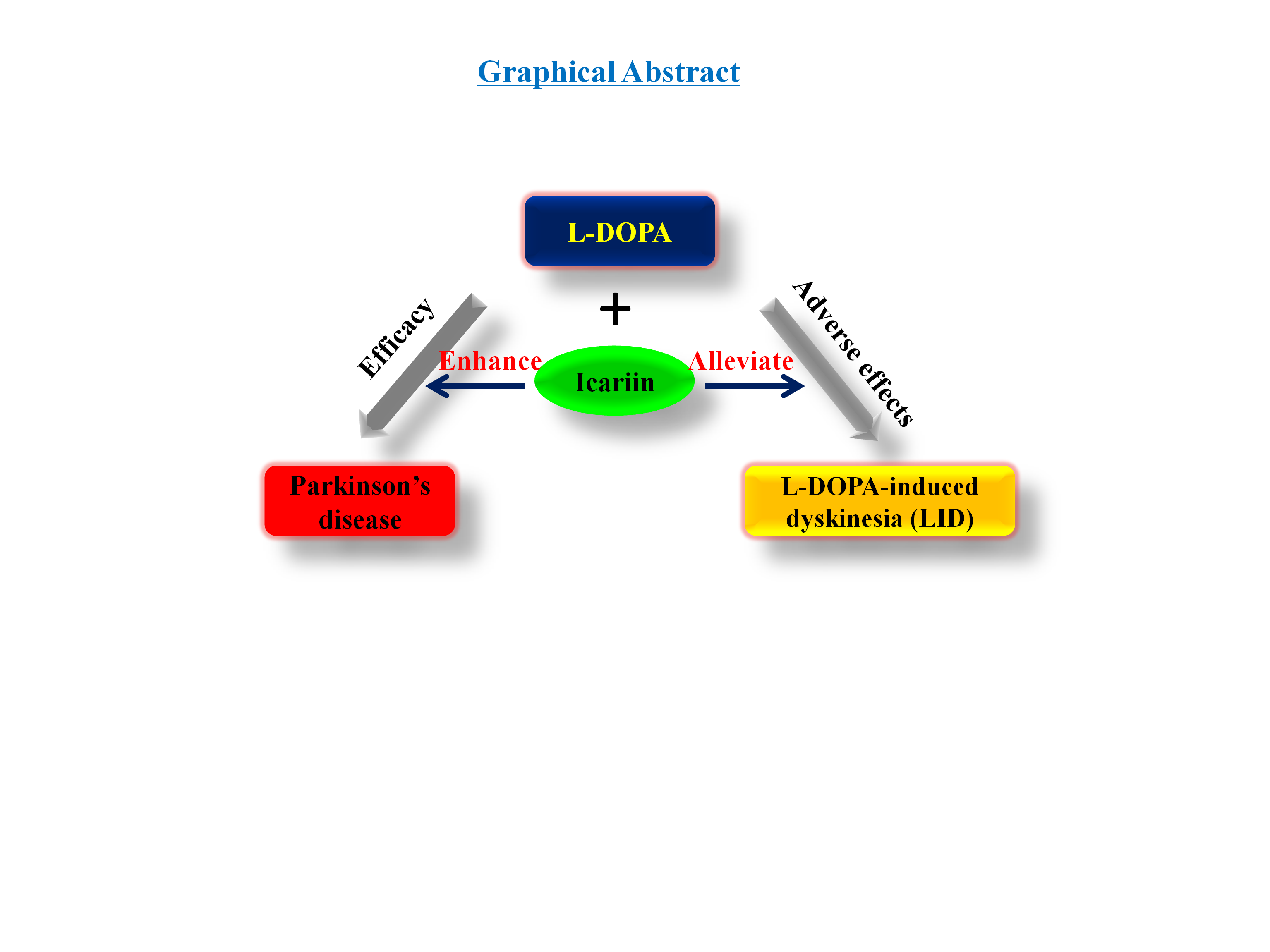

Supplement: FIGURE S1 — Icariin enhances L-DOPA efficacy and attenuates L-DOPA-produced adverse effects in Parkinson’s disease. [file Image_1.TIF]
